# Comparative analyses of nuclear proteome: extending its function

**DOI:** 10.3389/fpls.2013.00100

**Published:** 2013-04-26

**Authors:** Kanika Narula, Asis Datta, Niranjan Chakraborty, Subhra Chakraborty

**Affiliations:** National Institute of Plant Genome Research, Aruna Asaf Ali MargNew Delhi, India

**Keywords:** nucleus, comparative proteome, organeller proteome, plant, nuclear proteins

## Abstract

Organeller proteomics is an emerging technology that is critical in determining the cellular signal transduction pathways. Nucleus, the regulatory hub of the eukaryotic cell is a dynamic system and a repository of various macromolecules that serve as modulators of such signaling that dictate cell fate decisions. Nuclear proteins (NPs) are predicted to comprise about 10–20% of the total cellular proteins, suggesting the involvement of the nucleus in a number of diverse functions. Indeed, NPs constitute a highly organized but complex network that plays diverse roles during development and physiological processes. In plants, relatively little is known about the nature of the molecular components and mechanisms involved in coordinating NP synthesis, their action and function. Proteomic study hold promise to understand the molecular basis of nuclear function using an unbiased comparative and differential approach. We identified a few hundred proteins that include classical and non-canonical nuclear components presumably associated with variety of cellular functions impinging on the complexity of nuclear proteome. Here, we review the nuclear proteome based on our own findings, available literature, and databases focusing on detailed comparative analysis of NPs and their functions in order to understand how plant nucleus works. The review also shed light on the current status of plant nuclear proteome and discusses the future prospect.

## INTRODUCTION

Cell nucleus has a perplex, heterogeneous, self renewable, and dynamic social milieu which can sense signals, deformations, mechano-transduction, biochemical deliberations and many other processes ensuing outside its boundary. Nucleus is enclosed in a phospholipid rich membrane, which has very sensitive ion channels and pores that shuttles biomolecule in and out by conformational and morphological transformations. The nucleus, often referred as the “eukarya,” is functionally divided into the nuclear interior, a structurally differentiated and articulated organization surrounded by an envelope which is dynamic but sensitive to outside milieu (Dahl et al., 2008). In lieu, the modular disposition of indispensable, dynamic, and complex morphological feature of nuclear locale administers its function. It paves the way for the role of nuclear architecture in critical regulatory processes. The nucleus is a fundamental component of the microenvironment of both plant and animal cells that has been substantially expanded during evolution and keeps the genetic material separate from other activities of cell (Roix and Misteli, 2002). It is the ultimate exhilaration of gene regulation and proteins directly controlling the gene expression (Wilson and Dawson, 2011). Furthermore, it has been reported that the nucleus plays an important morpho-regulatory role during organogenesis in animal, besides its pivotal role in chloroplast division in plants (Cavalier-Smith, 2006; Dahl et al., 2008). The plant nucleus has biomechanical and morphogenetic functions; it is a viscoelastic solid encompass temerity of protein complexes. The organization of nuclear proteins (NPs) into versatile assemblies provides precise control over the shape, size, and composition of the nucleus, which opens a route toward the construction of sensors, programmable packaging and cargo delivery system within the sub-nuclear compartments as well as between the organelle. Plasticity in the nucleus allows cell differentiation, while rigidity in the nucleus determines its mechanical stiffness (Jiang et al., 2006; Pajerowski et al., 2007). Beyond its paramount importance in the generation of form, nucleus is frequently considered “growth-regulating” (Cavalier-Smith, 2006). The nucleus is evolutionary and inherently bestowed with information that can be both stored and relayed to cell interior via templating processes. It serves as the regulator in cell signaling for perceiving and transmitting extra- and inter-cellular signals in many cellular pathways. Communication between the cytoplasm and the nucleus is necessary and evident because of events such as apoptosis (Broers et al., 2002), mechanical stress (Dahl et al., 2008), environmental perturbation (Cheung and Reddy, 2012) and pathogen infection (Rivas, 2012), which lead to altered biosynthesis and modification of nuclear architecture and downstream cytoplasmic events. In addition, nucleoskeleton acts as a substrate for genome partitioning during mitosis. Further, it has been recognized as a central portal for providing motor centers during chromosome segregation in cell division (De Souza and Osmani, 2009). However, the available data is rather scarce and motor proteins between nucleoskeleton and chromosomes are still not known in higher plants. Throughout the plant kingdom the formation and regulation of the nuclear architecture has been shown to have the potential to influence many conduits of development, epigenetic differentiation, microfabricated patterning and cell senescence, besides environmental stress response and pathobiology (Vergnes et al., 2004; Constantinescu et al., 2006; Dahl et al., 2008). Also, the nucleus serves a multi-functional role, as a regulator and modulator during cell division, and controller and integrator for fertilization and inheritance. Thus, nucleus plays a critical role as a modulator of cellular phenotype (Franklin et al., 2011). The nucleus must therefore be dynamic as cells divide, modulating its composition and architecture during its formation and after it has been disintegrated. The nuclear function is a multi-step, complex process, and the underlying mechanisms governing these steps are not fully understood.

All eukaryotic lineages are characterized by the loss, gain, expansion, and diversification of gene families (Fritz-Laylin et al., 2010). Understanding protein diversity and shared features can give unprecedented insight into the most fundamental aspects of nuclear structure and protein organization in as diverse kingdoms as plants and animals. Determination of organellar proteomes – the complement of proteins that reside, even if temporarily, in a specific organelle or sub-cellular region is of fundamental importance. Sub-cellular fractionation of tissue and cells in combination with MS/MS analysis has proven to be a powerful approach for the identification of proteins contained in specific organelles, such as the nucleus. Proteome research holds the promise of understanding the molecular basis of the nuclear function using an unbiased comparative and differential approach. Although the field of angiosperm eukaryogenesis has plethora of contradictory ideas, the nature of molecular changes can be reflected from the proteome. The nuptials of proteomics with cell biology have produced extensive inventories of the proteins that inhabit several sub-cellular organelles, including nucleus (Schirmer and Gerace, 2005; Yates et al., 2005; Yan et al., 2008). We and others have identified several hundred plant and animal NPs that include both predicted and non-canonical candidates, presumably associated with a variety of functions; viz., nucleoskeleton structure, development, DNA replication/repair, chromatin assembly/remodeling, signal transduction, mRNA processing, protein folding, transcription and splicing regulation, transport, metabolism, cell defense and rescue; all of which impinge on the complexity of NPs in plant (Pandey et al., 2006; Choudhary et al., 2009) and animal (Henrich et al., 2007). In recent years, reports have also been published focusing on changes in the nuclear proteome in varied cellular events (Bae et al., 2003; Lee et al., 2006; Salzano et al., 2006; Henrich et al., 2007; Buhr et al., 2008; Pandey et al., 2008; Repetto et al., 2008, 2012; Choudhary et al., 2009; Abdalla et al., 2010; Cooper et al., 2011; Varma and Mishra, 2011; Abdalla and Rafudeen, 2012). The identified proteins revealed the presence of complex regulatory networks that function in this organelle. NPs have been shown to account for approximately one-fourth of total proteins in yeast (Moriguchi et al., 2005) and one-fifth in animals (Bickmore and Sutherland, 2002), but the arithmetic estimate in plants is not yet complete. Currently, the focus is on nuclear proteomes in order to understand the nucleus-related processes in plants and animals. Although over the past few years there have been rapid advances in nuclear proteome research, the study on the complexity of NPs remained secondary, despite the fact they correspond to about 10–20% of the total cellular proteins and are comprised of several hundred different molecules with diverse functions. Moreover, a vast array of post-translational modifications to these proteins add diversity to the structure and ligand-binding properties of nuclear components, leading to their differential activity. Therefore, characterization of the nuclear proteome in plant hold the promise of increasing our understanding about the regulation of genes and their function.

Here, we begin by giving updates on the nuclear proteomes and summarizing the essential and unique features of the nucleus. We also discuss recent findings concerning the regulation and biochemistry of it with specific emphasis on the fundamental role of NPs in development, DNA replication/repair, transcriptional regulation, environmental stress, and signaling by analyzing the nuclear proteomes. Furthermore, we report the cross-kingdom comparative analysis of nuclear proteomes toward organism specificity and plant exclusivity based on our own findings, the available literature and databases focusing on NPs in view of the current understanding and perspectives of the nuclear functions.

## ORIGIN OF THE NUCLEUS

A landmark event in the evolution of eukaryote was the acquisition of nucleus. Eukarogenesis have evolved albeit independently in plants and animals. Although both are true eukaryotes they have different ancestors. The evolution from prokaryotes to eukaryotes was the most radical change in cell organization. It is known that evolution of complex characters typically involves preadaptation, radical mutational innovation, and different selective forces acting in succession (for review, see Cavalier-Smith, 2006). Physical and mutational mechanisms of origin of the nucleus are seldom considered beyond the longstanding assumption that it involved wrapping pre-existing end membranes around chromatin (Cavalier-Smith, 1988). Evolution of the nucleus starts approximately 850 Million years ago (Cavalier-Smith, 2002), but it was 1833 when Robert Brown discovered the nucleus and said “vim and vigor is sexless devoid of this facet” in a paper to the Linnean Society. Origin of nucleus requires understanding of co-evolution of different nuclear components and their functional interlinking into the fundamentally novel eukaryotic life style. There are two competing theories of eukaryotic evolution. According to the first theory, a subset of bacteria slowly developed nucleus, while in the other, eukaryotes came first, some of them then lost nucleus and gave rise to bacteria. But, woesean revolution highlights that eukaryotes came from archaeal stock. Since, eukaryotes contain both archaeal and bacterial genes and the division of labor arose from the ancient symbiotic partnership between them that gave rise to eukaryotic nucleus. A third option for the nuclear origin revolves around the viruses, but the supporting data are provocative, circumstantial, and controversial (for review, see Pennisi, 2004).

## DESCRIPTION OF TOOLS TO STUDY NUCLEAR PROTEOME

**FIGURE 1:**
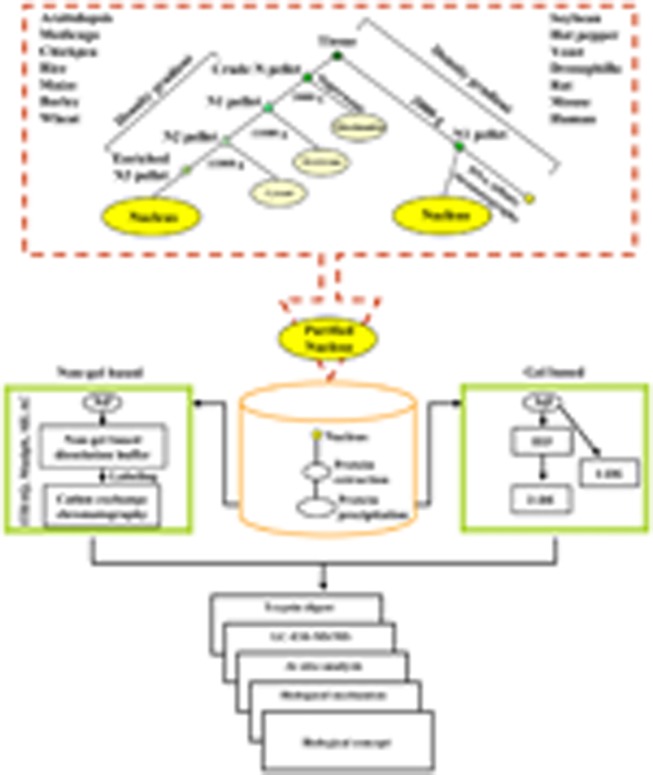
**A flowchart illustrating the overall experimental strategy for the analysis of the nuclear proteome.** NP denotes nuclear protein.

An outline of the procedure and the illustration of the data that can be generated with the methodology are shown in **Figure 1**. Each proteomic study is described through a simplified flowchart showing its different steps from experimental material to protein identification. As illustrated in **Figure 1**, density gradient methods can be used to prepare a nuclear fraction with or without DNA affinity chromatography. The most efficient means to separate NPs are either two-dimensional gel electrophoresis or cation-exchange chromatography followed by elution of protein fractions with salt gradient. Over the past few years 2-DE coupled MS/MS and LC-MS/MS have extensively been used to study nuclear proteomes in varied organisms (for reviews, see Khan and Komatsu, 2004; Cullen and Mansuy, 2010; Erhardt et al., 2010). In brief, the NP fractions after separation are digested to allow identification of proteins by mass spectrometry. Proteins can be directly submitted to enzymatic digestion with appropriate proteases, such as trypsin or to chemical treatment to get peptides of appropriate mass (usually between 750 and 4000 Da). Identification of proteins can then be done either by peptide sequencing using liquid chromatography coupled to MS (LC-MS/MS) or by peptide mass mapping using matrix-assisted laser desorption/ionization-time of flight (MALDI-TOF/TOF) followed by *in silico* analyses. Custom NP databases, for example, yeast-NPD^1^, human-NPD^2^, *Medicago*-NPD^3^, TAIR^4^, rice nuclear proteome database^5^ help improve the identification and post-translational modification of the NPs. To undertake a comprehensive comparison of the plant nucleolar proteomes based on a combined approach of alignment, structure and phylogeny an *Arabidopsis *nucleolar protein database was curetted (Brown et al., 2005). Similarly, comprehensive and well-annotated database of transcription factors may provide a useful resource to check annotations and to study gene regulatory pathways (Guo et al., 2005, 2008; Iida et al., 2005; Gao et al., 2006; Palaniswamy et al., 2006; Zhu et al., 2007; Rushton et al., 2008; Perez-Rodriguez et al., 2010; Romeuf et al., 2010; Wang et al., 2010). 

Nuclear proteins are often under-represented in proteomic studies due to their low abundance. The information offered from total nuclear proteome by high-throughput techniques does not illustrate the functional purpose of NPs and compartment structures. Computational modeling, on the other hand, may elucidate functional roles otherwise not captured by any individual existing experimental technology. The predictions used to identify the common organelle-specific sequence features are successful for over-represented proteins but is limited for the low abundant proteins. Thus, this analysis allows the identification of additional proteins sharing the same motif and to estimate the enrichment of the protein motifs in nuclear proteome data set. The estimation of the enrichment of those motifs in the nuclear proteome data set was done by comparing their frequencies in the nuclear data sets and in the target protein databases. Some of the motifs that can be identified in the nucleus are domains of well-known proteins, including histones and helicases. There are many proteins that are known to be imported into the nucleus, but which have no known intra-nuclear compartment association. These proteins may share similar cellular locations or functions, but further experiments are needed for clarification (Gorski and Misteli, 2005).

## WHAT HAVE WE LEARNT?

Proteomics has turned out to be an imperative benefactor for studying the acquaintance of plant nuclear structure and function. The field of proteomics is evolving from cataloguing the proteins under static conditions to comparative analyses (Narula et al., 2012). Defining proteins that change in abundance, form, location or other activities may indicate the presence and functional significance of a protein. Whereas comparative nuclear proteome research is quite advanced in animals (Liao et al., 2009) and yeast (Gauci et al., 2009), there is less information in plants. The investigation on plant nuclear proteomes in recent years has raised the following important questions: What are the essential plant NPs? Do NPs show clade specificity in vascular plants? What are those organ-specific NPs, if any? Does the nuclear developmental proteomics of one of the clades yield any astonishing or prolific results? How do NPs remodel during environmental- and/or patho-stress those provide new perspectives? Are some of the NPs unexpected? And, last but not the least, what sort of post-translational modifications have so far been characterized in the nucleus? Here, we analyze and compare the experimental results thus far available on nuclear proteomes to elucidate the dynamics of plant NPs.

## DECIPHERING THE ORGANISM-SPECIFIC NUCLEAR PROTEOME DYNAMICS: SOCIAL CLASS VS. DIVERSITY

Proteins evolve at rates differing over many orders of magnitude. As new proteins evolve by gene duplication, evolutionary rates must change dramatically over time. They change systematically among different branches of the evolutionary tree and also episodically (Cavalier-Smith, 2010). In the history of life there are three mega evolutions giving rise to prokaryotes, plants, and animals. Consequently, decoding organism specific nucleoid/nuclear proteomes are of utmost importance to understand the diversity among the protein complement in the three life lines of fruition. The possibility of intra-kingdom and/or cross-kingdom comparison of proteins and cellular regulation with the use of advanced proteomic techniques are of great value. We compared the experimentally determined nuclear proteomes of plants viz., *Arabidopsis thaliana*, *Cicer arietinum, Medicago sativa*, *Glycine max*, *Capsicum frutescens*, *Xerophyta viscose*, and *Oryza sativa* with that of yeast, fruit-fly and animal (human, rat, and mouse; **Table 1**). The *modus operandi* in investigating the nuclear proteomes of available species were the extensive literature search, availability of relevant databases (human, mouse, rat and plant, TIGR, UniProt and Swissprot) and *in silico *analysis. After comparing individual NPs, we investigated the divergence of these proteins among animals and plants to understand the integration and coordination of nuclear functions. Further, we calculated the percentage of proteins that was found to be unique to each proteome by calculating the number of proteins estimated from matches to SWISS-PROT as described in Skovgaard et al. (2001), Semple et al. (2003), and Bhushan et al. (2006). The NPs identified in these studies were classified into different functional categories. This classification is only tentative, since the biological role of many of the proteins identified has not been established experimentally. Furthermore, we applied a cross-species comparison on the available datasets. When analyzing proteomes within the specified group of plants, a logical strategy was used to maximize efficiency and the overall comparative results. Thus, it was imperative to first evaluate the available nuclear proteome maps, followed by an analysis of stimulus-specific proteomes of the above mentioned organisms. We then moved on to assess the stress-responsive plant nuclear proteomes in order to understand the overlap and specificity amongst different environmental- and patho-stress. These comparative studies were customized for specific protein families. It is to be noted that protein consensus can be obtained across any combination of proteomes based on the type of extraction procedure.

**Table 1:** A comprehensive list of plant nuclear proteomes.

S. No.	Organism	No. of proteins identified	Biological material used for fractionation	Technique used	Experimental conditions	Reference
1	*Chlamydomonas reinhardtii*	672	Cell suspension culture of algae	nanoLC-MS/MS	Cell	Winck et al. (2011)
2	*Arabidopsis thaliana*	154	Leaves	2DE/LC-MS/MS	cold	Bae et al. (2003)
3	*Medicago truncatula*	143	Seed	nano-liquid chromatography–nano-LC-MS/MS	Development	Repetto et al. (2008)
4	*Cicer arietinum*	150	Leaves	2DE/LC-MS/MS	Tissue	Pandey et al. (2006)
5	*Cicer arietinum*	150	Leaves	2DE/LC-MS/MS	Dehydration	Pandey et al., 2008
6	*Xerophyta viscosa*	18	Leaves	2-DE/ MALDI-TOF-TOF	Dehydration	Abdalla et al. (2010)
7	*Xerophyta viscosa*	121	Leaves	2-DE/ ESI- MS/MS	Dehydration	Abdalla and Rafudeen (2012)
8	*Glycine max*	292	Leaves	nano-liquid chromatography–nano-LC-MS/MS	Rust fungus	Cooper et al. (2011)
9	*Capsicum annuum*	56	Leaves	2-DE/MALDI-TOF-MS	Tobacco mosaic virus	Lee et al. (2006)
10	*Linum usitatissimum*	292	Immature seed coat at 16daf (Torpedo stage)	1-DE, WB, Dot blot, gel shift	Development	Renouard et al. (2012)
11	*Oryza sativa*	468	Endosperm	LC-MS/MS	Tissue	Li et al. (2008)
12	*Oryza sativa*	109	Leaves	2DE/LC-MS/MS	Dehydration	Choudhary et al. (2009)
13	*Oryza sativa*	307	Leaves	Percoll gradient centrifugation-nano LC-MS/MS	Glucose responsive	Aki and Yanagisawa (2009)
14	*Zea mays*	Not identified	Endosperm	1-DE, WB	Development	Ferreira et al. (2006)

**FIGURE 2:**
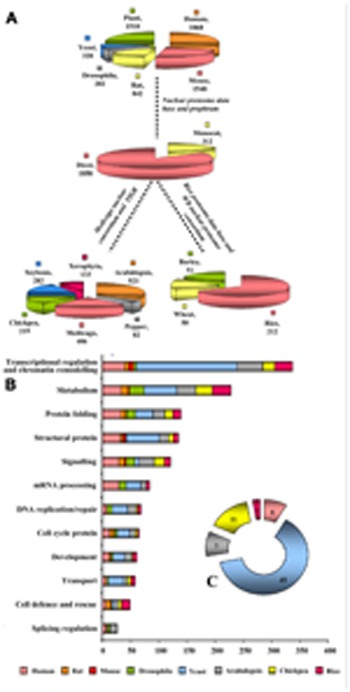
**Benchmarking nuclear proteomes.** **(A)** Cross-kingdom-clade-species comparison of nuclear proteomes. **(B)** Functional classification of the identified proteins was made according to the biological processes. The length of the bar indicates the number of proteins present in a particular species, such as, *Arabidopsis*, chick pea, rice, human, rat, mouse, *Drosophila*, yeast*. ***(C)** The pie chart inset represents the fraction of unknown proteins in**each of these organisms.

Nuclear protein composition was found to differ between two major Kingdoms viz., plant and animal. Results were predominantly obtained with human (lymphoma and myeloid lines; Salzano et al., 2006; Henrich et al., 2007), mouse (Buhr et al., 2008), rat (McClatchy et al., 2011), *Drosophila* (Varma and Mishra, 2011), model plants *Arabidopsis *(Bae et al., 2003), *Medicago*(Repetto et al., 2008, 2012), and crop plants rice (Choudhary et al., 2009), hot pepper (Lee et al., 2006), soybean (Cooper et al., 2011), *Xerophyta* (Abdalla et al., 2010; Abdalla and Rafudeen, 2012), and chickpea (Pandey et al., 2006, 2008). The stunning findings from these comparisons suggest that until now only 1868 NPs are identified in humans, while 1548 in mouse, 842 in rats, 282 in *Drosophila*, 328 in yeast, and 1510 in plants contributing to the large repertoires of the nuclear proteome database and prophram (**Figure 2A**). A more accurate vision of animal nuclear proteome illustrate that approximately 2000 NPs from lymphoid and myeloid tissues of humans symbolizing around one-third of their estimated nuclear proteome. The two branches of angiosperm, monocot, and eudicot, firmly set up compelling evidence from the available nuclear proteomes that some NPs are unique, while some are shared. Extended nuclear proteome research is required for monocot family with only 312 NPs reported thus far than dicots that reported 1856 NPs (**Figure 2A**). Contextual information on nuclear proteomes of eudicots revealed that until now 521 NPs have been identified in *Arabidopsis *representing about one-third of its estimated nuclear proteome (Bae et al., 2003), while 406, 282, 219, 82, and 133 NPs were identified in *Medicago*, soybean, chickpea, hot pepper, and *Xerophyta*, respectively. To explore early messages arising from comparison of the content of monocot and dicot proteomes address key consequences of research for dicot comparative proteomics. The recurring observation that monocot proteome research centers on rice proves factual for nuclear proteome. Until recently, 212 NPs have been identified in rice, whereas only 50 and 51 NPs are known in wheat and barley, respectively (**Figure 2A**). Our comparative analyses of different species in relation to their function showed that high percentage of proteins to be unique to each proteome: 89% in animal (human, rat, and mouse), 81% in human (lymphoma and myeloid lines), 71% in mouse, 68% in rat, 84% in yeast, and 74% in *Drosophila*; whereas plant proteomes show 85% in *Arabidopsis*, 78% in soybean, 81% in chickpea, 71% in *Medicago*, 84% in rice, and 54% in hot pepper with only actin and 26S proteasome being the social class of proteins present ubiquitously in all. The available nuclear proteomes of nine plants compared in **Figure 2A** varied in molecular weight from 9.1 to 150 kDa and had a spread of p*I *values from 3.6 to 10; while yeast shows 15 to 110 kDa, 3.1 to 12.0 pI; *Drosophila* shows 12 to 140 kDa, 3.1 to 11.4 pI and animals show 9.4 to 150 kDa, 3.0 to 12.0 pI. Most of the NPs were basic in nature concordant with the acidic environment of this organelle.

Functional categorization of nuclear proteomes reported till date revealed an imperative corollary, which shows overall proteins belonging to transcriptional regulation and chromatin remodeling contribute radically to nuclear proteomes of yeast (54%), plants (29%), animals (16.5%), and *Drosophila* (1.2%). 54% of proteins in this category in yeast represent more than half of the total NP implying thereby that yeast is a dynamically dividing organism having plurality of transcription regulators. Of these, 42% represents nuclear structural proteins involved in cell division. Furthermore, plants are deskbound, therefore to establish, maintain and alter global and local level of nucleic acid they require rapid turnover of DNA and RNA metabolizing proteins, DNA replication/repair proteins, and splicing regulation proteins toward acclimatization in the environment. Indeed, identified plant NPs from the available reference proteomes showed 42% NPs belong to metabolism category whereas 46% confers splicing regulation. NPs behave like a network scaffold and acts as an entry point to ensure smoother regulation of different cellular processes that require rapid protein turn over. Comparison of plant nuclear proteomes with other organisms revealed that protein folding and turnover category contributes to 35%, which is in close correlation to 30% NPs from animals. Plant protein networks revealed the predominance of the development specific proteins (36%) and cell cycle proteins (24%). It is to be noted that the NP extraction protocol used in the animal and plant are different. Animal nuclear proteome research spotlight sub-nuclear compartmentalization, whereas, plant nuclear proteomes except *A. thaliana *(Calikowski et al., 2003; Pendle et al., 2005) and* O. sativa* (Tan et al., 2007, 2010) tranquil total nucleus. Therefore, the chromatin assembly/remodeling proteins are identified much less in all organisms as they can be isolated best in high salt buffer concentration which is not usually used for total NP extraction. A dramatic rearrangement of the nuclear structure takes place during mitosis and meiosis, which dynamically changes sub-nuclear proteomes (Beven et al., 1995; Holmes-Davis and Comai, 1998). Major role of interphase chromatin is in transcription, while mitotic chromatin contributes to cell division and meiotic chromatin is engaged in pairing, cross-over, and chromosome segregation. Compositional divergence in protein complement of stationary and dividing nucleus is thus a call to study proteomes at different phases of nuclear division.

Between plant and animal, the gene families and members are not related but functionally appear to be similar. Mosaic comparison of nuclear proteomes revealed that chimeric evolution was the main cause of proteome diversity in animals. For example, in human nucleophosmin protein has diverse protein members, whereas in mouse it is conserved. Likewise, when rat and mouse were compared DEAD, septin, lamin B2 box are some of the diverse class of proteins found in rodents. Also, when human, rodents and *Drosophila* were evaluated lactate dehydrogenase, nicotinamine synthase1, tubulin beta, and 26S proteasome represented the social class. Furthermore, when, human, rodent (mouse, rat), insect (*Drosophila*) and fungus (yeast) were compared actin and nicotinamine synthase 1 correspond to the social class. Whereas, comparison between monocots and dicots, showed that *Arabidopsis* is better explored than rice and therefore comparison of their proteomes may not yield the postulated results as defined by genome analysis. But the proteins revealed an evolutionary divergence in plant as well as dicot vs. monocot specificity, with few conserved proteins (**Figure 2B**). When the *Medicago* nuclear proteome was compared with that of *Arabidopsis*, results revealed an evolutionary divergence as well as tissue specificity, with few conserved proteins (**Figure 2B**). Comparison of the functional classes of NPs amongst dicot species like *Arabidopsis*, *Medicago*, soybean, hot pepper, *Xerophyta*, and chickpea confirm the dynamic and heterogeneous nature of nucleus as exemplified by the presence of only actin in all dicots. Another protein namely 26S proteasome may be considered as social class except its absence in *Xerophyta*. The presence of chaperone 60 in *Cicer *and HSP71in *Medicago* illustrate that nature invented vastly different solutions to a common problem viz., protein folding. When the studies on the legumes like *Cicer* and *Medicago *were compared to *Arabidopsis* belonging to the Brassicaceae family (**Figure 2**), it can be readily observed that the splicing regulation in the nucleus for activating splicing enzymes is diverse between the two families as well as between the members of the same family, leguminosae. The protein network of rice revealed the predominance of the chromatin assembly/remodeling proteins, for example, histone deacetylase, histone 2A, histone 2B, histone 3, histone 4, while the *Arabidopsis* protein network was found to be rich in splicing regulation proteins and structural protein as transcriptional regulators.

It may be assumed that the divergence in the resulting proteomes of the vascular plants is due to the presence of the different nuclear architecture based on the protein and nucleic acid compositions, suggesting the occurrence of clade-specific NPs that would bind to their cognitive biomolecules to bring out specific functions both spatially and temporally. Most intriguing are the remaining 10–18% of plant NPs that do not have any similarity to the known proteins in other organisms. The challenge is to elucidate their biological role within the cell nucleus.

## EXPLORING THE SINK AND LINK IN NUCLEUS

Ubiquitously present, except in RBC, the nucleus is composed of different molecules with diverse functions to meet the specialized requirements of different organs and tissues. Nuclear functional compartmentalization is a paradigm of molecular machines necessary for biogenesis and functionality (Strouboulis and Wolffe, 1996). It is a dynamic milieu having a reservoir for bioactive molecules, such as carbohydrates, nucleic acid, and proteins which is necessary for assembly and also for communication with the other parts of the cell. For decades, cell nucleus has been a black box in biology. The determination of comprehensive chemical differences between plant and animal nucleus is still difficult to understand, but the switching of cellular programs by NPs mediated chemical networking is tightly linked to the regulation of gene expression in both the kingdoms. However, the distributions of transcription sites in chromosome territories are conserved in plants and animals. It is the heterochromatic centers which makes the difference in nuclear processes in both these kingdoms (van Driel and Fransz, 2004). Being a store house of nucleic acid and proteinaceous domain, nucleus contains distinct structural and functional compartments (Misteli, 2005). Proteinaceous domain include nucleolus containing rRNA binding protein and splicing proteins, the cajal body having snRNA forming and binding proteins; whereas the nucleic acid domain encompass euchromatin and heterochromatin. Euchromatin is the reservoir of histones and histone binding proteins, while heterochromatin consists of heterochromatin binding unknown proteins. In these two domains nucleic acid occurs three dimensionally (Dundr and Misteli, 2001). Nuclear bodies are functionally and/or morphologically discrete accommodating usually distinct resident proteins. Paradigm includes the nucleoli (site of rRNA transcription), nuclear speckles (site for splicing) and splicing factor compartment (store-house for cajal body and PML body; Takizawa and Meshorer, 2008). Dramatic developments in high-resolution live-cell imaging have revealed the cell nucleus as a highly heterogeneous and complex organelle, and the global genome and proteome architecture changes during processes such as differentiation and development (Misteli, 2001; Spector, 2003; Misteli, 2005). It is, therefore, relevant that different family members show highly regulated and specific patterns of the expression of nuclear components in an evolutionary context. Similarities in nuclear design may be apparent as it is likely that ancient functional protein domains and nucleic acid backbones have been used in a variety of arrangements and combinations to affect the function of convergent biological structures. Nucleus serves as the self organizing mediator. Most proteins are in constant motion, and their residence time within a compartment is very low, being at most 1 min (Gonzalez-Melendi et al., 2000). This mobility ensures that proteins find their targets by energy-independent passive diffusion (Pederson, 2000). In addition to protein heterogeneity and the presence of various regulators, mediators, transducers as well as linkers, RNA and chromatin compositions can vary between cell types and even within a given cell in different time (Hetzer et al., 2005), suggesting that the nucleus serves as a sink of variability in terms of macromolecules or microelements. Regulated trafficking of proteins, RNAs, RNA-protein complexes, and other molecules in and out of the nucleus is important in diverse processes. The nucleus serves as the end line culminator in cell signaling to perceive and transmit extra- and intercellular signals in many cellular pathways. NPs not only constitute more than just a structural scaffold, but also play various roles in development, cell cycle, defense against environmental stresses and in the tight regulation of gene expression.

## THE NUCLEAR PROTEIN SINK: A DYNAMIC FRAMEWORK FOR MULTIPLE FUNCTIONS

Eukaryotic NPs are complex with plurifunctional role, evolutionary tinkering, and subtle modifications evoked repeatedly and independently among different taxa. A macromolecular machine in the form of nuclear pore allows a protein or protein complex up to approx. 500 kDa to traverse the nucleus. NPs roam through the nucleus in search of a high-affinity binding site where they can exert their functions (Dundr and Misteli, 2001; Mans et al., 2004). The specific domain and architecture of NP contain information of biological importance and evolutionary value.

Altogether, NPs include those which are highly mobile viz., transcription factors, pre-mRNA splicing factors, rRNA processing enzymes and 3α-processing factors, DNA repair enzymes, chromatin-binding proteins and apoptotic caspases; while immobilized NPs encompass DNA replication factors, intermediate filament proteins, and histones H1 (Dundr and Misteli, 2001). Plant and animal show least homology as far as nuclear intermediate filament proteins are concerned. However, proteins belonging to DNA replication/repair are found to be orthologous (Moriguchi et al., 2005). In plants, elongation factor thermo unstable (EF-Tu), Zinc finger protein, glycine rich RNA binding, histone 2B, histone 3, glycine dehydrogenase, peptidyl prolyl isomerase, 26S proteasome, 60 kDa chaperone, glyceraldehyde 3-phosphate dehydrogenase, malate dehydrogenase, peroxiredoxin, transaldolase, calcium protein kinase, PHO1 like protein, ά expansin, actin, 14-3-3, and 40S ribosomal protein SA are consistently represented in thus far studied nuclear proteomes that play diverse and crucial roles in nuclear function. Most predominant class of NPs reported are the transcription regulators in which TF2A, RNA polymerase have been optimized during eukaryotic evolution for acting in post-transcriptional gene regulation. The linear representation of promoter elements provides competency for physiological responsiveness within the contexts of development, cell cycle, and phenotype-dependent regulation as transcription factors can bind to these cis-acting elements dictating where and when a gene to be active. Chromatin binding proteins and nucleosome organization protein viz., PolII, MADS box, RCC2 protein, and HEAT box reduce distances between independent regulatory elements providing a basis for integrating components of transcriptional control. It is known that the nuclear matrix proteins support gene expression by imposing physical constraints on chromatin related to the three-dimensional genomic organization. In addition, the nuclear matrix proteins facilitate gene localization besides the concentration and targeting of transcription factors. Histone deacetylase 6 and DNA methyltransferase physically interact; together they mediate histone acetylation and modulate DNA methylation status, silencing the transposable element (Casati et al., 2008; Casati, 2012). Transcriptional reprograming by WRKY, ERF, TGA, Whirly, and MYB factors is thought to cause alteration in transcript level, which in turn regulates various physiological processes like growth, development, and pathogen perturbation (Mayrose et al., 2006; Yu et al., 2011; Feng et al., 2012). Among others, it is ascertain that G5bf protein and TF rough sheath 2 are embodied persistently in dicot nuclear proteome in customary environment. Perhaps, the protein most expected to be similar to their metazoan counterpart in the plant nucleus is DNA ligase, which have been shown to regulate transcription (Truncaite et al., 2006). RF2B, SPT2-chromatin binding domain, RING zinc finger protein, and gypsy-like retroposon are exclusively present in monocot nuclear proteomes (Li et al., 2008; Aki and Yanagisawa, 2009; Choudhary et al., 2009). Aforesaid, transcription regulators of two clades have solitary similarity that they are regulated by circadian rhythm and have multivariate decision to find motif combination (Cavalier-Smith, 2010). Proteome data indicate that BABY BOOM, AP2/EBEBP2, and syringolide induced proteins are leguminosae allied transcriptional regulators having role in development, cell/organ identity and fate; while ribosomal recycling factor, CHP rich zinc finger protein, nucleolin, RuvB, BRI KD interacting protein, WPP domain protein, pescadillo protein, and MYB transcription factor are solanaceae associated transcriptional regulators (Boutilier et al., 2002; Abe et al., 2003). Each of these proteins reported in leguminosae and solanaceae have been shown to be involved in diverse cellular functions, viz. development, embryogenesis, and signaling pathways. This further highlights the technical challenges when attempting to isolate high purity nucleus and resolution of proteins using proteomic technology. Proteins involved in the metabolism are customary in the case of any nuclear proteome. Indeed, methylenetetrahydrofolate reductase, homocysteine methyl transferase, methyltransferase, and ornithine aminotransferase are proteins belonging to this category, which play pivotal role in RNA and DNA metabolism. Astounding result obtained from the comparative analysis of nuclear proteomes in plants with that of animals, suggests the presence of many metabolism related animal orthologs in dicot (*Arabidopsis*) proteome whose role in plants have not yet been defined viz., biliverdin reductase A1, LROS1 acyl transferase, and KES1 oxysterol binding protein. Nuclear structural proteins are ubiquitous in both the kingdom but shows more divergence in plants (Meagher et al., 1999). Actin and myosin are the ancient component of this category that form a platform for all three DNA-dependent RNA polymerases, mediate RNA export from the nucleus, and are required for the long-range movement of specific loci within the nucleus (Caudron-Herger and Rippe, 2012). Recent evidence suggests that proteins such as actin, myosin, tubulin, NuMA, Annexin A1, Annexin A2, viscialin, spectrins, and titin are recognized as having fundamental roles in nuclear structure and genome function in living eukaryotes (Wilson and Dawson, 2011). Coiled-coil protein, an orthologue of lamin, the building block of the NPC complex in plants (Mans et al., 2004) has many candidates, namely disease resistance proteins, NUF1, NUP82, NUP88. Nucleoporins, which anchor intermediate filament proteins during scaffold formation play a crucial role in chromosome scaffolding and mRNA export. The nucleoskeleton and nucleopore complex protein present ubiquitously in plants are expansins and NUPs. They maintain the mechanostatic and load bearing properties of the nucleus (Dahl et al., 2008). In other words, nucleostructural dynamics in plant cell is a team effort of multiple proteins orchestrating this very fast-paced game. Nucleus is an evolutionary chimera of cell cycle related proteins. Progress made in the area of plant cell cycle regulation has resulted in recognition of NP candidates, including chromobox proteins, RCC2 proteins, and BUB3 having role in cell division. Additionally, CDC 5, one of the cell cycle proteins has a varied role in mitosis, ciliary motility and trafficking. Another component of cell cycle regulation namely, Ran cycle is represented by nuclear Ran GTPase, mago nashi protein, Ras GTPase, and Ran. Ribosome subunit export system of nucleus involved in cell cycle regulation focus on the three most appealing candidates: Nops, Nugs, GTPases, besides recently added AAA-ATPase and exportin in animals. However, analyses of plant nuclear proteomes do not show the presence of these proteins. Perhaps another protein most expected to be similar to their metazoan counterparts in the plant cell nucleus is karyopherins, which has a role in nuclear trafficking. Nucleus includes numerous enzymes viz., rRNA processing enzyme, polymerase, ligase, gyrase, and number of helicase that alter DNA conformation, replication, degradation, and chromatin modifiers. Histone variants, one of the important classes of NPs in eukaryotes are important component which play a key role in genome maintenance and stability. In the nucleus, RNA is an architectural factor for shaping the genome and its nuclear environment, besides being an effector molecule in maintaining the chromatin structure (Ma et al., 2011). We find many mRNA processing proteins that include nucleolar RNA-associated protein (NRAP), LSM2, paraspeckle protein 1, and Non-POU domain containing protein in this category. It is well known that protein folding supports diverse but specific signal transducers and lies at the interface of several developmental pathways (Caudron-Herger and Rippe, 2012). Likewise, different chaperones, HSP71, proteasome subunit alpha types, DnaJ, protein disulfide isomerase, HSP20, glutathione-S-transferase, and HSP70 reported in plant nuclear proteomes might maintain protein homeostasis by providing stability to other nuclear resident proteins. Involvement of some of these chaperones with the class of developmental NPs viz., DEAD box, DUX3, von wilberand factor, HOMEO BOX, and U box have already been reported (Barthelery et al., 2008; Su and Li, 2008). A chronic theme proverbial to the class of nucleoskeleton linker proteins of plant cells is that these mechano-transducing transmembrane molecules communicate and interact preferentially with the intermediate filament on the nuclear side of the nuclear membrane. Our analyses suggest, several attributes of NP contribute to cross-talk in gene regulation and cellular phenotype.

## NUCLEAR INVENTORIES FOR *IN SILICO* PROTEIN PROFILING OF COMPARATIVE STRESS PROTEOME

Nucleus senses and physiologically responds to environmental stress via signaling pathways. Signaling events are clearly not linear and induce many different reactions, including stress-related processes that crosstalk with hormone signaling pathways. Most signaling pathways culminate in the nucleus leading to regulation of expression of specific genes whose products are necessary for eliciting a signal specific response like nuclear localization of pathogen effectors, R proteins, and other host defense proteins that modulate stress response. Here, we have customized the comparative analyses for specific protein families. For example, when the environmental stress-responsive proteomes were compared, the parallel analysis of the proteomes of different clades of vascular plants were performed, viz., chick pea vs. *Xerophyta* vs. rice for dehydration, *Arabidopsis* for cold response, and *Medicago* for seed filling that mimic the dehydration response. Similarly, in case of patho-stress, soybean, and hot-pepper proteomes were compared.

**FIGURE 3:**
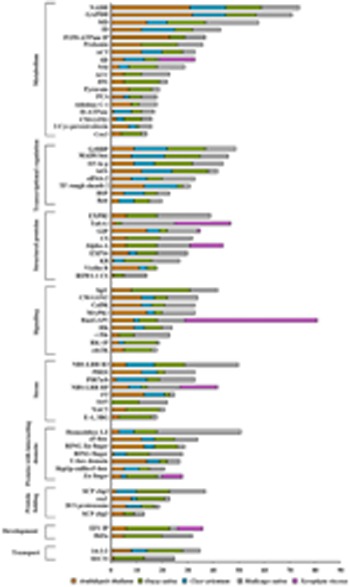
**Comparative abiotic stress-responsive nuclear proteome: The functional classification of the identified proteins was according to the biological processes in which they are involved.** The length of the bars indicates the number of proteins present in a particular stress.

We analyzed the nuclear proteomes of *A. thaliana* in response to cold- stress (Bae et al., 2003) and dehydration-responsive nuclear proteomes of *Cicer* *arietinum* and *O.* *sativa* (Pandey et al., 2008; Choudhary et al., 2009). Interestingly, a great level of divergence in the protein classes amongst these organisms was observed (**Figure 3**). To our surprise, except development category all of the NPs were found to have members common in all organisms under both kind of abiotic stresses studied. Families of development related proteins, viz., embryonic flower 1- like protein, copia-like, and Hd3a protein have been found in dehydration responsive proteome of rice, while chickpea DRPs exclude most of the nuclear structural proteins such as cellulose synthase like, alpha amylase, and beta-expansin otherwise abundantly present in dehydration-responsive proteome of rice. It is intriguing to note that cold-responsive NPs under all functional categories of *Arabidopsis* were present in dehydration-responsive proteomes of rice and chickpea. Another important finding was the presence of cyc3 protein in high abundance during cold-stress in *Arabidopsis*. Whereas zinc finger, ring finger, and RNA glycine rich proteins were predominantly found during dehydration response but were absent in response to cold-stress. Various kinases known to mediate the stress-induced synthesis of NPs, such as PHO1, galectin, thioredoxin peroxidase were present both in monocot and dicot under varied stresses. Our analyses revealed the presence of monocot and dicot cdc-2k, SEC31, TubA1 having specific protein sequences that clearly demonstrate the diversity of the identical NPs in two divisions of angiosperm. This may be attributed to the evolution of orthologs vs. paralogs.

**FIGURE 4:**
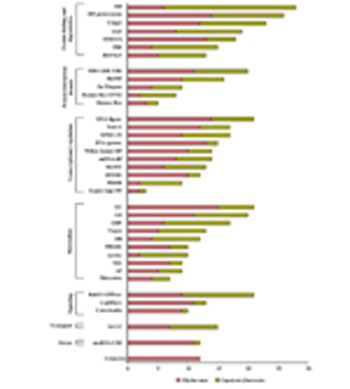
**Comparative patho-stress responsive nuclear proteome: The functional classification of the identified proteins was according to the biological processes in which they are involved.** The length of the bars indicates the number of proteins present in a particular stress.

Responses to various patho-stresses largely depend on the plant’s capacity to modulate rapidly but specifically its proteome. External signals are translocated into the nucleus in a stress-type dependent manner to activate transcription factors, resulting in the increased expression of particular sets of defense-related genes. During evolution, mutual recognition between plants and pathogens has resulted in development of fascinating variety of molecular strategies in the nucleus of the host against the invader. Some pathogens have been shown to directly activate transcription (Lev et al., 2005). It is now well accepted that modulation of chromatin configuration is an additional strategy employed by pathogen to subvert plant immune response (Ma et al., 2011). Nevertheless, plants also dispose an array of proteins in the nucleus that act as a scrutiny scheme to allow the early detection of an impending pathogen assault. We analyzed the nuclear proteomes of soybean and hot pepper in response to fungal (Cooper et al., 2011), and viral (Lee et al., 2006) stresses, respectively (**Figure 4**). The widespread NPs identified in fungal and viral stresses belong to the category of protein folding and degradation. On the contrary, it was interesting enough to observe that there was not a single protein to be exclusive in case of either soybean-rust interaction or hot pepper–tobacco mosaic virus (TMV) interaction. During these host–pathogen interactions complex architecture of nucleus might respond differently against two different pathogens but using same set of NPs. Fungal stress and viral stress both might induce fundamental machinery of the nucleus to correctly target expressed proteins in a diverse but adaptation- related pathway thereby barricade the pathogens. However, NPs belonging to protein folding and degradation, transcription regulation, and metabolism categories toward patho-stress needs further consideration to understand the fungal-viral difference or specificity.

## CONCLUSION

Since its existence was first discovered almost 180 years ago, the nucleus has been a central focus of biological research. Initially it was assumed that nucleus is a static organelle. Progress over the years has gradually changed this view, and more recently, the importance of the NPs in chromatin organization, gene regulation, and signal transduction has become evident. In this study, cross-kingdom, cross-species as well as cross-condition comparisons of nuclear proteomes in vascular plants and animals illustrates the divergence in protein profiles within only a few social classes. *In silico *experimental analyses of the nuclear interior revealed a morphologically structured yet dynamic mix of NPs. Major nuclear events depend on the functional integrity of protein species and their timely interaction. Yet, unknown drivers of protein ensure that they are in the right place at the time when they are needed. Furthermore, the incessant unrest of proteins can be captured by the comparative nuclear proteome study under various regulatory events. As expected, the proteins involved in transcriptional regulation and chromatin remodeling were found to be the most predominant across all conditions. Nonetheless, a large number of proteins were unique or novel to each of the clades and under different stresses. It may be thought, the ubiquitously present protein classes are essential for sustenance, while the unique classes bring out the condition-specific special function. The differences in terms of protein pattern and protein function appear to encompass both genetic and physiological information. It may be speculated that the differential proteome is shaped by the cellular environment and the ecological niche of the corresponding organism. The divergence may arise due to codon bias, amino acid composition, and protein length. A much more comprehensive survey of the nuclear proteomes in several plants will ultimately draw a more complete picture of the social class vs. protein diversity in this organelle.

## MARCHING AHEAD: NEXT FIVE YEARS

We are witnessing a significant but inadequate progress in understanding the nuclear proteomes of various crops of agricultural importance. Our understanding of nuclear composition, organization, and homeostasis has been greatly enhanced through targeted biochemical and genetic approaches. Unbiased “discovery” methods, such as proteomics, have only recently gained traction in the field of regulation biology. To date, a key word search using “Plant nuclear proteome” retrieves only 116 results in a pubmed search, emphasizing the need for in-depth study in the field. Although our knowledge of nuclear proteome and NPs has greatly increased, many open ended questions remain to be answered. It is to be noted that few thousands NPs identified in the nuclear proteomes have not been functionally characterized. Thus, for this new and emerging field, we predict that the potential for an accelerated pace of future discoveries in nuclear cell biology is tremendously high. The future scientific interest should center around the diverse roles NPs play in regulating cell division, growth, differentiation, aging, disease, and environmental perturbations. The comparative analysis of organism, clade-specific and stress-responsive plant nuclear proteomes revealed the presence of certain proteins that were unexpected, either in their abundance, form, number or else localization. These unexpected or non-canonical proteins suggest the constant remodeling of nuclear proteomes. The exact function and specificity of these candidates can only be comprehended once they are functionally characterized. Furthermore, role of PTMs on gene expression and NP-interactome dynamics remains as two important but challenging facets. Our future efforts will focus on the development and analysis of comparative nuclear proteomes toward an understanding of crop- and genotype-specific adaptation as an important amendment for the determination of protein networks influenced by the internal and external cues associated with the complex cellular, biochemical and physiological process that bring about phenome variation.

## Conflict of Interest Statement

The authors declare that the research was conducted in the absence of any commercial or financial relationships that could be construed as a potential conflict of interest.

## Footnotes

1 www.pin.mskcc.org

2 www.npd.hgu.mrc.ac

3 www.masc.proteomics-org

4 www.arabidopsis.org

5 http://gene64.dna.affrc.go.jp/RPD
